# Effect of large dosage of Fuling on urinary protein of diabetic nephropathy: A protocol of systematic review and meta-analysis of randomized clinical trials

**DOI:** 10.1097/MD.0000000000022377

**Published:** 2020-10-02

**Authors:** Jia Xia, Li Zhang, Xinxia Zhang, Lizhen Wang, Botong Yang, Qi Chen, Min Zhong, Xiaoming Tang, Jun Zhou

**Affiliations:** a Hospital of Chengdu University of Traditional Chinese Medicine; b Chengdu University of Traditional Chinese Medicine, Chengdu, Sichuan, China.

**Keywords:** Fuling (Poria cocos), large dosage, diabetic nephropathy, meta analysis and systematic review, protocol

## Abstract

**Introduction:**

Diabetic nephropathy (DN) is one of the most common and serious microvascular complications in patients with diabetes, which seriously affects their life quality and survival time. large dose herb Fuling or compound prescription contain large dose Fuling for treatment of DN has already been confirmed. However, due to the lack of evidence, there is no specific method or suggestion, so it is necessary to carry out systematic evaluation on Fuling and provide effective evidence for further research.

**Methods and analysis:**

The following databases will be searched from their inception to June 2020: Electronic database includes PubMed, Embase, Cochrane Library, Web of Science, Nature, Science online, Chinese Biomedical Database WangFang, VIP medicine information, and China National Knowledge Infrastructure (CNKI). Primary outcomes:24-hurinary-albumin, Urinary albumin-to-creatinine ratio. Additional outcomes: Serum creatinine, Blood urea nitrogen, Glomerular filtration rate, Endogenous creatinine clearance rate. Data will be extracted by 2 researchers independently, risk of bias of the meta-analysis will be evaluated based on the Cochrane Handbook for Systematic Reviews of Interventions. All data analysis will be conducted by data statistics software Review Manager V.5.3. and Stata V.12.0.

**Results:**

The results of this study will systematically evaluate the effectiveness and safety of large dose Fuling intervention for people with DN.

**Conclusion:**

The systematic review of this study will summarize the current published evidence of large dose Fuling for the treatment of DN, which can further guide the promotion and application of it.

**Ethics and dissemination:**

This study is a systematic review, the outcomes are based on the published evidence, so examination and agreement by the ethics committee are not required in this study. We intend to publish the study results in a journal or conference presentations.

**Open Science Framework (OSF) registration number:**

August 24, 2020. osf.io/ym2c6. (https://osf.io/ym2c6).

## 1 Introduction

Diabetic nephropathy (DN) is a syndrome characterized by persistent proteinuria and progressive decline of renal function,^[1]^ which is the leading cause of end-stage renal disease worldwide.[^2^,^3^,^4^] In recent years, due to the aging of the population and the high incidence of diabetes, the number of patients has been increasing year by year,^[5]^ which has brought a heavy economic and health burden to individuals and society. At present, DN is as high as 20% to 40% in the primary disease of end-stage renal disease, a serious disease.^[6]^ DN is an important primary incidence, and can also lead to related cardiovascular diseases.

In terms of treatment, the current clinical western medicine for the treatment of DN is still symptomatic treatment, such as regulation of blood pressure, blood sugar, blood lipid, etc. The selected drugs are usually limited and often accompanied by adverse reactions.^[7]^ At the same time, due to the variety of drugs taken, poor patient compliance, fear of progressive decline in renal function, and common symptoms of most DN patients, such as frequent nocturnal urination, oliguria, limb swelling and cold limbs. Therefore, even though pure western medicine treatment can accurately and quickly deal with the abnormal clinical biochemical indicators, there is no specific method for improving symptoms and improving patients’ quality of life.

In recent years, traditional Chinese medicine has been widely used in clinical and experimental study of DN, which had been fully proven effective. Fuling is botanical herb commonly used in traditional Chinese medicine and the exploration of DN treatment methods show that large-dose Fuling had good effects on reducing urine protein and improving DN symptoms, but its effectiveness and safety have not yet reached a definitive conclusion. Therefore, this research intends to adopt the method of system valuation and meta analysis of large-dose Fuling or contain large-dose Fuling prescription in the treatment of DN to evaluate the efficacy and safety.

## 2 Methods

### 2.1 Study registration

The protocol has been registered in Open Science Framwork (OSF) Preregistration. August 24, 2020. osf.io/ym2c6. (https://osf.io/ym2c6). The protocol will follow the statement guidelines of Preferred Reporting Items for Systematic Reviews and Meta-Analyses Protocols (PRISMAP),^[8]^ changes will be reported in the full review as required.

### 2.2 Inclusion and exclusion criteria for study selection

#### 2.2.1 Inclusion criteria

Inclusion criteria are all randomized controlled trials (RCTs), which treatment of DN is large dose Fuling or Fuling is main element in mixture herb formulas. The language of the trials to be included only Chinese or English.

#### 2.2.2 Exclusion criteria

Following studies will be excluded:

1. patients age <18 years old
2. other types of kidney diseases. Such as IgA nephropathy, Lupus nephropathy, and so on.
3. the treatment was combined with other treatment other than Chinese herbs.
4. Non-RCTs and Quasi-RCTs
5. Case series and Reviews
6. Animal studies.

### 2.3 Types of participants

Types of participants included people diagnosed with DN, no matter the degree. All the patients should be treated by traditional Chinese medicine included Fuling, or Fuling is main element in mixture herb formulas, or the herb combines with other conventional treatments. No sex, ethnicity, or education restrictions is here.

### 2.4 Experimental interventions.

The traditional Chinese medicine Fuling should be the main treatment. Dosage limits of the herb should be: large dose Fuling (more than 60 g, 4 times larger than the maximum prescription dose of *the Pharmacopoeia of the People's Republic of China* 2015 version).

### 2.5 Control interventions.

Interventions may include: The placebo, nondrug interventions (eg, diet, exercise, etc), conventional western medicine treatment (such as oral hypoglycemic drugs, antihypertensive drugs, lipid-regulating drugs, etc). Combined interventions are allowed as long as all groups in the randomized trial receive the same combined intervention.

### 2.6 Types of outcome measures

#### 2.6.1 Main outcomes

1. 24-hurinary-albumin;
2. Urinary albumin-to-creatinine ratio.

#### 2.6.2 Additional outcomes

1. Serum creatinine;
2. Blood urea nitrogen;
3. Glomerular filtration rate;
4. Endogenous creatinine clearance rate.

## 3 Data sources

### 3.1 Electronic searches

The following data bases will be searched to identify eligible studies: PubMed, Embase, Cochrane Library, Web of Science, Nature, Science on line, Chinese Biomedical Database WanFang, VIP medicine information, and China National Knowledge Infrastructure (CNKI). The time range is: the starting time is determined according to the first literature available, and the deadline is August 2020.

### 3.2 Other search resources

**Figure 1:**
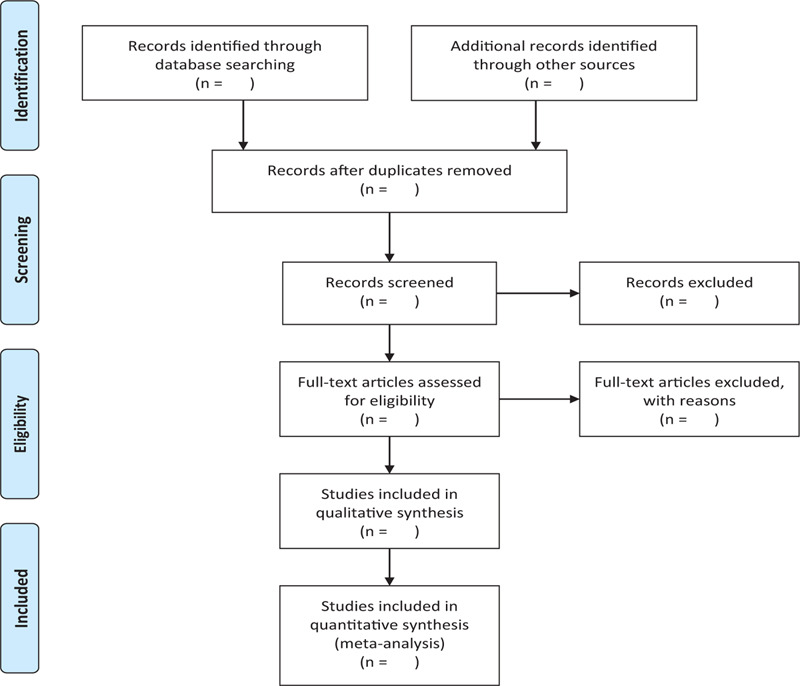
The research flowchart. This figure shows the identification, screening, eligibility, and included when we searching articles.

In order to get more complete evidence, we will also retrieve other related documents by manually, such as medical textbooks, clinical laboratory manuals and so on. If it is necessary, we will contact with trail author to obtain the latest clinical data. Moreover, studies associated with the review will be identified via evaluating related conference proceedings. The research flowchart is shown in Figure 1.

### 3.3 Search strategy

The following search terms will be used: randomized controlled trial/RCT; Diabetic nephropathy/DN; Traditional-Chinese-Medicine/TCM; Fuling/Fu-Ling/Poria-cocos. Different retrieval strategies in Chinese and foreign databases will be used. Language restrictions are Chinese and English. There is no publication restriction. Here we take the search strategy in PubMed as an example and list in Table 1.

**Table 1:**
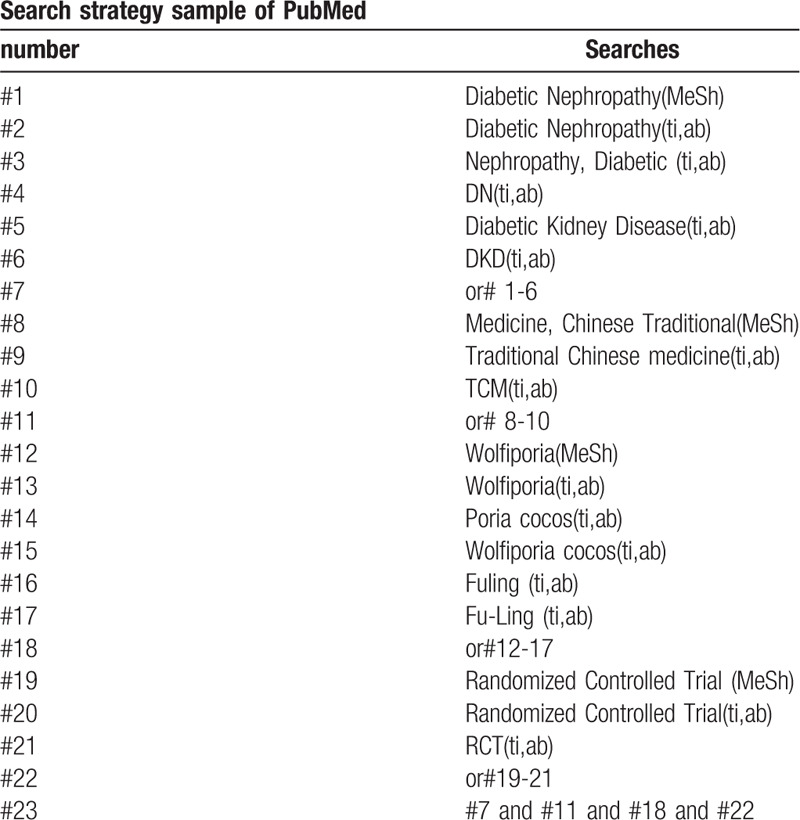
The research strategy.

## 4 Data collection and analysis

### 4.1 Study selection

All articles in the search results were independently evaluated by 2 independent researchers (JX, LZ) according to inclusion and exclusion criteria. Reviewers will then independently extract and collect the data included in the study using predesigned data collection forms. Discrepancies will be discussed and resolved by consensus with a third author (XZ).

### 4.2 Data extraction and management

The following information will be extracted from each study:

1. Normal test characteristics: title, author, year.
2. baseline data: sample size, age, gender, diagnostic criteria, course of disease.
3. interventions: dosage of Fuling, control of intervention details, intervention.

If the information is not enough, we will contact experts and authors in this field to get relevant information.

### 4.3 Assessment of the reporting quality and risk of bias

The risk of bias will be assessed by 2 independent authors (JX and LZ), together with completing the STRICTA checklist.^[9]^ The Cochrane System Evaluator's Manual give the evaluation criteria for authors to evaluated the RCTs’ quality. Assessing the risk of bias:

1. random sequence generation;
2. allocation concealment;
3. blinding of participants and personnel;
4. blinding of outcome assessment;
5. incomplete outcome data;
6. selective outcome reporting;
7. other bias.

Any disagreement will be discussed or consulted with a third reviewer. Each them will be described from three levels: “high risk,” “low risk” and “unclear.”

### 4.4 Measures of a treatment effect

The dichotomous outcomes will be expressed by the odds ratio, while the continuous data will use the standardized mean difference. All these outcomes report 95% confidence intervals.

### 4.5 Management of missing data

We will take the method of contacting corresponding authors to obtain the missing data. If there is no response, we will analyze only the available data and describe the reason and impact of this exclusion in the paper.

### 4.6 Assessment of a reporting bias

The bias of publication will be explored through funnel plot analysis. If the funnel plot show asymmetry, it will be evaluated via the Egger and Beg tests, and *P* value <.05 means the publication bias is significant.

### 4.7 Assessment of heterogeneity

There are 2 main methods for testing heterogeneity, namely graphical method (funnel plot, forest plot) and statistical test (*Q* value statistic test, *I* ^2^ statistic test, H statistic test). The *I* ^2^ statistic test method shows us when *I* ^2^ is 0, it means that studies are completely homogeneous. If *I* ^2^ > 50%, it indicates there is heterogeneity in studies.

### 4.8 Data synthesis and grading of quality of evidence

The results of the study will be analyzed by RevMan 5.0 software provided by Cochrane collaborate on network. The binary data will be expressed by the odds ratio, while the continuous data will use the mean difference. To test the heterogeneity of the research results, when the *I* ^2^ < 50% or *P* > .1, the heterogeneity is significant. The random effect model was used for the meta-analysis, otherwise, we choose the fixed effect model.

### 4.9 Subgroup analysis

 

### 4.10 Sensitivity analysis

Sensitivity analysis cannot only assess the stability and reliability of the conclusions of the meta analysis, but also assess whether the changes in the results are related to the impact of a single study. If the stability of the conclusion is poor, we can achieve when the heterogeneity test results are heterogeneous, we need to clarify the source of the heterogeneity by subgroup analysis. The effects of different types of therapy including design scheme, severity of illness, age, sex, and mild or severe DN were analyzed. We will also delete low-quality and/or medium-quality studies to check the robustness of the results.

The purpose of increasing stability by changing the analysis model, inclusion and exclusion criteria, or excluding a certain type of literature.

### 4.11 Ethics and dissemination

We will publish the system review results in peer-reviewed journals, disseminated in meetings or in peer-reviewed publications. Aggregated published data will be used to exclude data of individuals, so there is no need for obtaining the ethical approval or patients’ informed consent.

## 5 Discussion

Fuling is a traditional Chinese medicine for strengthening spleen and kidney, benefiting water and dampness. It can be used for a variety of metabolic diseases, such as obesity, hyperlipidemia, coronary heart disease, DN, etc. Modern pharmacological studies[^10^,^11^,^12^] have shown that Fuling has anti-fibrosis, anti-tumor, anti-inflammation, anti-oxidation, anti-aging, regulating immunity, and other functions.

The chemical constituents of Fuling mainly include polysaccharides, triterpenes and sterols,^[13]^ which have extensive pharmacological activities and rich targets.^[14]^ The treatment of DN mainly includes the following characteristics:

1. Fuling polysaccharides mainly consists of 1-3-β-D polysaccharides, which can promote the expression of Klotho protein, protect endothelial cells from senescence pathway, and delay hyperglycemia endothelial damage;
2. Pachyman has the effect of inhibiting chronic renal interstitial fibrosis and delaying the progression of renal failure;
3. Existing in vivo and in vitro experiments^[15]^ showed that, as one of the representative compounds of Fuling, poria acid could not only exert its anti-inflammatory effect by down-regulating the expression of inflammatory mediates such as IL-1, IL-6, and TNF-α in cardiomyocytes, but also significantly reduce the postprandial blood glucose level of diabetic mice by enhancing insulin sensitivity.^[16]^ It can even reduce the expression of proteins and mRNA related to the formation of extracellular matrix in mice to reduce the damage of renal tissue, so as to achieve the purpose of kidney protection;^[17]^
4. Activate Fox O1 to inhibit the mesenchymal transformation of epithelial cells in the hyperglycemia state, improve the pathological features of “proteinuria” in diabetic mice and reduce renal damage.

Doctor of traditional Chinese medicine has always been refined to consider the herbs’ dosage. The standard dosage of Fuling in *The Pharmacopoeia of the People's Republic of China* (2015 edition) is 10 to 15 g. However, in clinical practice, Chinese physicians have their own unique cognition of the specific dosage of Fuling. Taking Zhang Zhongjing's *Treatise On Febrile Diseases* and *Synopsis Of Golden Chamber* as examples, the range of one-time application of Fuling is very wide, ranging from 1.25 g to 40 g. The dosing of Fuling was divided into small dose (<10 g), medium dose (10–20 g) and large dose (>20 g). The most frequently used volume of Fuling was 10 to 20 g, accounting for 52% of the amount of Fuling prescription in Zhang Zhongjing decoction.^[18]^ The application of Fuling in small dose has a poor clinical effect in the treatment of some diseases or acute critical diseases. The reason using large dosage to reach treatment effection is that Traditional Chinese medicine mostly plays a role in a single herb, commonly by the form of compound herbs or prescriptions. Clinical experience confirms that the dosage of Fuling and its compound preparation is generally larger. Chunfeng et al^[19]^ treated heart failure of cardiomyopathy with Zhenwu Decoction (Fuling 100 g), with an effective rate of 97.13%. In addition, the property of Fuling is gentle, and no adverse reactions were reported in large dose use.^[20]^ Therefore, it is also suggested that it is necessary to consider Chinese herbs rather than chemical extracts in clinical medication, and the dosage should be more in line with clinical practice, so as to accurately reflect the functional value of traditional Chinese medicine and its compound.

In conclusion, the systematic review and meta-analysis are helpful to determine the potential value of high-dose Fuling and the herb combination therapy for DN. To improve the quality of life in severe patients. This study can not only provide the basis for the release of DN treatment guidelines, but also promote the application of traditional Chinese medicine prescriptions, so that more patients benefit.

## Author contributions

**Conceptualization:** Jia Xia, Li Zhang, Qi Chen, Xiaoming Tang, Xinxia Zhang.

**Data curation:** Li Zhang; Lizhen Wang, Xinxia Zhang, Min Zhong, Xiaoming Tang.

**Formal analysis:** Jia Xia, Min Zhong, Xinxia Zhang.

**Methodology:** Jia Xia, Li Zhang, Lizhen Wang, Xiaoming Tang.

**Project administration:** Lizhen Wang, Xinxia Zhang.

**Resources:** Jia Xia, Botong Yang, Qi Chen.

**Software:** Botong Yang, Qi Chen, Jia Xia, Li Zhang, Jun Zhou.

**Supervision:** Qi Chen, Xinxia Zhang.

**Writing–original draft:** Jia Xia

**Writing-review & editing:** Xinxia Zhang.

## References

[1] Nagib AM Elsayed Matter Y Gheith OA Diabetic nephropathy following posttransplant diabetes mellitus. Exp Clin Transplant 2019;17:138–46. 30945628 10.6002/ect.2018.0157

[2] Zhang L Long J Jiang W Trends in chronic kidney disease in China. N Engl J Med 2016;375:905–6. 27579659 10.1056/NEJMc1602469

[3] Duran-Salgado MB Rubio-Guerra AF Diabetic nephropathy and inflammation. World J Diabetes 2014;5:393–8. 24936261 10.4239/wjd.v5.i3.393 PMC4058744

[4] Yu XB Yu YZ Pathogenesis and treatment progress of diabetic nephropathy. Chin New Clin Med 2017;10:1022–5.

[5] Zhang XH Cao SL Progress in the pathogenesis of diabetic nephropathy. *Med Rev*. 2009;25:1212–16.

[6] Yang W Lu J Weng J Prevalence of diabeteas among men and women in China. N Engl J Med 2010;362:1090–101. 20335585 10.1056/NEJMoa0908292

[7] Xie QF Liang GT Duan JH Advances in the treatment of diabetic nephropathy with Traditional Chinese medicine. China Med Herald 2019;16:46–9.

[8] Shamseer L Moher D Clarke M Preferred reporting items for systematic review and meta-analysis protocols (PRISMA-P) 2015: elaboration and explanation. BMJ 2015;349:g7647–17647. 10.1136/bmj.g7647 25555855

[9] John Wiley & Sons Ltd, Fellow JPHSSV Altman DG Assessing Risk of Bias in Included Studies. 2008.

[10] Zhang HR Lu HX Kang X Effect of Poria Cocos Polysaccharide on renal interstitial fibrosis in rats with type 2 diabetic nephropathy. Contemp Med 2016;22:1–2.

[11] Liang ZJ Research on the Regularity of Prescriptions in Ancient Books and Literatures of Traditional Chinese Medicine for Diabetic Nephropathy. Guangzhou, China: Guangzhou University of Traditional Chinese Medicine; 2014.

[12] Kong FJ Study on the Rule of TCM Syndrome Treatment of Diabetic Nephropathy. Beijing, China: Beijing University of Chinese Medicine; 2016.

[13] Xu S Jiang WQ Kuang YM Advances in the study of chemical constituents and biological activities of Poria cocos. Northwest Pharm J 2016;31:327–30.

[14] Zhang N Li ZX Li J Advances in the study of chemical constituents and biological activities of Poria cocos. *World Sci* , 2019, 21(2):220–33.

[15] Li F Yuan Y Liu Y Pachymic acid protects H9c2 cardiomyocytes from lipopolysaccharide -induced inflammation and apoptosis by inhibiting the extracellular signal-regulated kinase1/2 and p38 lightning pathways. Mol Med Rep 2015;12:2807–13. 25936656 10.3892/mmr.2015.3712

[16] Li TH Hou CC Chang CL Anti-hyperglycemic properties of crude Extract and Triterpenes from Poria Cocos. Evid Based Alternat Med 2011 2010 128402. 10.1155/2011/128402 PMC2949581 20924500

[17] Wang W Xu CG Zhou XJ Effect of poria cocos acid on interstitial fibrosis of obstructive hydronephrosis. Chi J Exp Surg 2014;31:2251–4.

[18] Li M He QY Chen YF Zhang zhongjing's study on the relationship between volume and effect of poria coiling. Chin J Tradit Chin Med 2015;30:4311–3.

[19] Jin CF Lv BL Zhao JD Therapeutic effect of zhenwu decoction reusing poria cocos on 174 cases of heart failure with cardiomyopathy. Chin Clin 2013;41:66–7.

[20] Jin Q Cao J Wang SH Pharmacology and clinical application of large dose tuckahoe. Zhejiang J Tradit Chin Med 2003 410–1.

